# Effects of Non‐Soy Legumes on Body Weight and Body Composition: A Systematic Review and Meta‐Analysis of Randomized Controlled Trials

**DOI:** 10.1002/fsn3.71365

**Published:** 2026-01-08

**Authors:** Reza Rahmanian, Mohsen Shaygantabar, Azita Hekmatdoost, Ali Nikparast, Fatemeh Javaheri‐Tafti, Zeinab Ghaeminejad, Andisheh Khoshrang, Alireza Hatami, Mohsen Mohammadi‐Sartang

**Affiliations:** ^1^ Department of Clinical Nutrition, School of Nutrition and Food Sciences Shiraz University of Medical Sciences Shiraz Iran; ^2^ Student Research Committee, School of Nutrition and Food Sciences Shiraz University of Medical Sciences Shiraz Iran; ^3^ Department of Clinical Nutrition and Dietetics, Faculty of Nutrition Sciences and Food Technology, National Nutrition and Food Technology Research Institute Shahid Beheshti University of Medical Sciences Tehran Iran; ^4^ Department of Nutrition, Faculty of Medicine Mashhad University of Medical Sciences Mashhad Iran; ^5^ Student Research Committee Mashhad University of Medical Sciences Mashhad Iran

**Keywords:** body composition, body weight, legume, meta‐analysis

## Abstract

Non‐soy legumes are rich in protein, fiber, and phytochemicals that may contribute to the prevention and management of obesity. Previous reviews have not specifically evaluated non‐soy legumes or comprehensive body composition outcomes, highlighting the need for a focused meta‐analysis. This systematic review and meta‐analysis aimed to evaluate the effects of non‐soy legume consumption on body composition parameters in adults, based on evidence from randomized controlled trials. Comprehensive searches were conducted in PubMed, Scopus, Medline, Web of Science, Cochrane Library, and Google Scholar up to January 2024 to identify eligible randomized controlled trials assessing the effects of non‐soy legumes on body weight, body mass index (BMI), waist circumference (WC), and fat mass (FM). Weighted mean differences (WMDs) and 95% confidence intervals (CIs) were calculated using a random‐effects model. Heterogeneity, subgroup analyses, meta‐regression, sensitivity analyses, and publication bias were evaluated using standard statistical methods. Thirty‐six trials met the inclusion criteria. Non‐soy legume consumption significantly reduced WC (WMD: −1.61 cm; 95% CI: −2.06, −1.16; *p* < 0.001), FM (WMD: −2.00 kg; 95% CI: −2.24, −1.78; *p* < 0.001), and body weight (WMD: −0.98 kg; 95% CI: −1.63, −0.33; *p* = 0.003). No significant effect was observed for BMI (WMD: −0.24 kg/m^2^; 95% CI: −0.50, 0.03; *p* = 0.08). Subgroup analyses indicated that the effects varied according to intervention duration, participant age, and baseline BMI. Non‐soy legume consumption is associated with significant reductions in body weight, waist circumference, and fat mass, but not BMI. These findings support the potential role of non‐soy legumes in improving anthropometric measures related to obesity, although further high‐quality trials are needed to confirm these effects.

## Graphical abstract

Consumption of non‐soy legumes significantly reduces body weight (−0.98 kg), waist circumference (−1.61 cm), and fat mass (−2.00 kg) in adults, according to a meta‐analysis of 36 randomized controlled trials. No significant effect was observed on BMI. These findings suggest that incorporating non‐soy legumes into the diet may be an effective dietary strategy for improving body composition and reducing obesity‐related anthropometric measures.

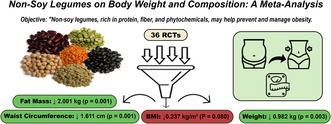

## 1 Introduction

Obesity is a significant public health challenge and is a main contributor to the development of various obesity‐related diseases and disabilities (Consultation WHO 2000). The global burden of obesity study suggested that due to the rising trend of obesity rates, about 1.12 billion people will experience obesity by 2030 (Kelly et al. 2008). Obesity is caused by a complex interplay of genetic, physiological, environmental, psychological, social, economic, and political elements, all of which contribute to its development (Aronne et al. 2009). Obesity is associated with a range of negative effects, including heart disease, type 2 diabetes, depression, all‐cause mortality, and decreased life expectancy (Consultation WHO 2000; Pischon et al. 2008; Bjørge et al. 2008).

Diet composition, as a modifiable determinant, is one of the most important environmental factors that contribute to obesity (Astrup and Brand‐Miller 2012). Recent comprehensive syntheses have strengthened the evidence supporting legumes as a protective dietary component against weight gain. An umbrella review of systematic reviews and meta‐analyses published in 2025 demonstrated that higher intakes of whole grains, legumes, nuts, and fruits were consistently associated with a lower risk of overweight and obesity, whereas greater consumption of red meat and sugar‐sweetened beverages was linked to a higher risk (Kristoffersen et al. 2025). These findings underscore the contribution of legumes to healthy body‐weight maintenance. However, most available evidence has been derived from observational studies, providing limited insight into causality and into specific anthropometric outcomes such as waist circumference or fat mass.

Non‐soy legumes encompass a wide variety of beans, including navy beans, chickpeas, kidney beans, garbanzo beans, and lima beans, as well as various types of peas, such as green peas and lentils. Non‐soy legumes are rich sources of plant‐based proteins, dietary fiber, phytochemicals, polyunsaturated fatty acids, and minerals such as potassium, calcium, and magnesium (Duranti 2006). Beyond their nutritional value, evidence suggests that non‐soy legume consumption is associated with favorable metabolic and cardiometabolic outcomes, including improved inflammatory profiles (Salehi‐Abargouei et al. 2015; Mansouri et al. 2024) and reduced risk of metabolic disorders (Thorisdottir et al. 2023; Bazzano et al. 2011).

In addition to these metabolic benefits, several randomized controlled trials have evaluated the effects of legume intake on body weight and adiposity. The most comprehensive quantitative assessment to date is the systematic review and meta‐analysis by Kim et al. (2016), which synthesized evidence from 21 randomized controlled trials involving 940 participants (Kim et al. 2016). The analysis demonstrated that diets incorporating dietary pulses including beans, lentils, chickpeas, and dry peas led to a modest but statistically significant reduction in body weight compared with control diets, even when total energy intake was not restricted. However, that study did not differentiate between soy and non‐soy legumes, and it did not include recently published RCTs, warranting an updated synthesis focused specifically on non‐soy legumes.

Despite the growing evidence supporting the metabolic benefits of legumes, research specifically isolating the effects of non‐soy legumes on anthropometric indices remains scarce. Previous meta‐analyses have typically combined soy and non‐soy legumes or focused solely on body weight, without examining other measures of body composition such as BMI, waist circumference, and fat mass. Moreover, several recent randomized controlled trials have evaluated non‐soy legume interventions but have not yet been quantitatively synthesized. Considering their distinctive nutritional composition and potential influence on energy balance and adiposity, a focused evaluation of non‐soy legumes is warranted. Therefore, this systematic review and meta‐analysis aimed to comprehensively assess the effects of non‐soy legume consumption on body weight, BMI, waist circumference, and fat mass in adults, thereby providing updated and robust evidence on their role in body‐weight regulation and metabolic health. To our knowledge, this is the first meta‐analysis to quantitatively pool randomized controlled trials addressing these specific outcomes, distinguishing it from earlier narrative reviews.

## 2 Materials and Methods

### 2.1 Study Protocol and Registration

This systematic review and meta‐analysis were conducted and reported in accordance with the Preferred Reporting Items for Systematic Reviews and Meta‐Analyses (PRISMA 2020) guidelines (Page et al. 2021). The PICOS framework was applied to define the research question and eligibility criteria (Table 1). The population of interest comprised adults (≥ 18 years), both healthy and unhealthy. The intervention was non‐soy legume consumption, while the comparator was a control or non‐legume diet. The primary outcomes were body composition parameters, including body weight, body mass index (BMI), fat mass (FM), and WC. The study protocol was registered in the International Prospective Register of Systematic Reviews (PROSPERO) (Registration No: CRD42025643508).

**TABLE 1:** PICOS criteria for inclusion and exclusion of studies.

Parameter	Criteria
Participant	Adults
Intervention	Fabaceae, bean, gram, faba, pea, lentil, lupin, legume, chickpea, pulses, non‐soy legume
Comparator	Control diet or non‐legume diet (e.g., meals without non‐soy legumes, usual or calorie‐restricted diets)
Outcomes	Weight, Body Mass Index (BMI), Waist circumference (WC), Fat Mass (FM)
Study design	Controlled trial

Abbreviations: BMI, body mass index; FM, fat mass; WC, waist circumference.

### 2.2 Search Strategy

Online databases inclusive of PubMed, Scopus, Web of Science, Cochrane Library, and the search engine Google Scholar were searched up to January 07, 2024, to find RCTs that assessed the effect of non‐soy legumes on body composition. The following Medical Subject Headings (MeSH) and non‐MeSH terms were searched within keywords, abstracts, and titles: “non‐soy”, “fabaceae”, “Vigna”, “Phaseolus”, “Castor Bean”, “ *Vicia faba* ”, “Abrus”, “Sophora”, “Sphenostylis”, “Pachyrhizus”, “Dipteryx”, and “Vicia”. The complete search strategy is provided in Appendix S1 (Table S1).

Additionally, reference lists of relevant systematic reviews and eligible studies were screened manually to identify additional trials.

All retrieved citations were imported into EndNote 20 for reference management and automatic duplicate removal. Given the large number of retrieved articles, a keyword‐based relevance filter was applied within EndNote to exclude clearly irrelevant publications (e.g., non‐human studies, non‐dietary interventions, or unrelated plant species) before manual screening.

### 2.3 Study Selection

Two independent investigators (FJT and ZG) then screened the remaining titles and abstracts to identify potentially eligible studies according to predefined inclusion and exclusion criteria. The full texts of all potentially relevant articles were retrieved and assessed for eligibility. Any discrepancies between reviewers were resolved through discussion and consensus with a third investigator (RR).

The inclusion criteria were as follows: (1) Randomized controlled trials (parallel or crossover design); (2) Adult participants (≥ 18 years); (3) Intervention involving non‐soy legumes (beans, lentils, peas, chickpeas, etc.); (4) Outcomes reporting body composition indices (body weight, BMI, WC, or FM).

The exclusion criteria were: studies that (1) had no control group; (2) administered legumes in combination with other active ingredients not matched in the control; (3) reported duplicate data and published as conference abstracts, reviews, editorials, letters, observational research, and case reports; (4) reported insufficient information on the baseline or follow‐up of inflammatory markers.

### 2.4 Data Extraction

The RCTs' data were reviewed by two investigators (FJT and ZG) according to established inclusion and exclusion criteria. Key characteristics of eligible RCTs were extracted and tabulated using a spreadsheet (Table 2). These characteristics included the first author's last name, publication year, study location, sample size, gender, target population, mean age of participants, details of study design, type of intervention and control, as well as the dose and duration of legume consumption. In cases where data were unreported, the first and corresponding authors of the RCTs were contacted via email for additional information. The mean values and standard deviations (SDs) for the outcomes of interest were extracted at baseline, following the intervention, and/or for the change between baseline and post‐intervention. In studies with multiple intervention arms, two groups were created based on the doses or different control groups. For trials with multiple intervention arms, two separate comparisons were created based on dose or comparator, ensuring that participants were not double‐counted in the meta‐analysis. Reported outcomes were converted to common units across studies for consistency.

**TABLE 2:** Main characteristics of the included RCTs.

Author, year, location	Design	Gender*	Duration (week)	Sample size	Age	BMI	Type of intervention	Type of placebo	Dose (g/day)	Population
Celleno et al. (2007), Italy	Parallel	Both	4	60	33.9	25.9	*Phaseolus vulgaris* extract^a^	Control dietary supplement	NR	Overweight
Crujeiras et al. (2007), Spain	Parallel	Both	8	30	36	32.0	Energy‐restricted + non‐soy legume diet^b^	Energy restriction without legumes	54	Obese
Winham and Hutchins (2007), USA	Crossover	Both	8	29	45.9	27.4	Baked navy beans^c^	Canned carrots	95	Hypercholesterolemic adults
Lee et al. (2009), New Zealand	Parallel	Both	16	74	57.9	30.5	Lupin bread^d^	Control bread	160	Overweight and obese
Gravel et al. (2010), Canada	Parallel	Female	16	134	51.3	29.8	Pulses^e^	Meals without pulses	535 mL/day	Metabolic syndrome
Hodgson, Lee, Puddey, et al. (2010), Australia	Parallel	Both	16	88	57.9	30.5	Lupin bread^f^	Standard white bread	160	Overweight and obese adults
Venn et al. (2010), New Zealand	Parallel	Both	72	113	42	35.3ᵃ	Calorie‐restricted diet + pulses^g^	Calorie‐restricted diet	95	Overweight and obese adults
Weiße et al. (2010), Germany	Parallel	Both	6	56	43.8	25.9	*L. angustifolius* (lupin)	Casein	35	Hypercholesterolemia
Wu et al. (2010), China	Parallel	Both	9	101	38.6	28.6	*Phaseolus vulgaris* extract^h^	Microcrystalline cellulose capsule	3	Overweight and obese adults
Belski et al. (2011), Australia	Parallel	Both	48	131	46.6	31.5	Lupin flour bread, biscuits, pasta^i^	Regular bread, biscuits, pasta	NR	Overweight and obese adults
Hermsdorff et al. (2011), Spain	Parallel	Both	8	30	36	32.5	Legume‐based diet^j^	Legume‐restricted diet	22.8–33.5	Obese adults
Abeysekara et al. (2012a), Canada	Crossover	Both	8	108	59.7	27.5	Pulse‐based diet^k^	Regular diet	150 (dry weight)	Adults ≥ 50 years
Jenkins et al. (2012), Canada	Parallel	Both	12	121	59.5	30.6	Low‐GI legume diet	High‐wheat fiber diet	190	Type 2 diabetes
Bähr et al. (2013), Germany	Crossover	Both	8	33	52.7	29.8	Lupin protein isolate	Milk protein isolate	25	Hypercholesterolemic subjects
Turner et al. (2013), USA	Parallel	Both	4	20	46.9	30.6	1.5 cups beans/day	Standard high‐fiber diet	285	Healthy adults
Alizadeh et al. (2014), 2014, Iran	Parallel	Female	6	34	36.1	36.1	Hypocaloric diet with legumes	Hypocaloric diet without legumes	190	Obese adults
Luzzi et al. (2014), Italy	Parallel	Both	12	60	47.5	NR	*Phaseolus vulgaris* extract^l^	None	0.2	Overweight adults
Tonstad et al. (2014), USA	Parallel	Both	16	173	48.4	36.4	Cooked beans	Reduced carbohydrate diet	125	Obese adults
Bähr et al. (2015), Germany (A)	Crossover	Both	4	72	NR	26.3	Lupin proteins	Milk proteins	25	Hypercholesterolemic adults
Bähr et al. (2015a), Germany (B)	Crossover	Both	4	72	NR	26.3	Lupin proteins	Milk proteins + Arginine	25	Hypercholesterolemic adults
Frota Kde et al. (2015), Brazil	Crossover	Both	6	44	57	26.3	Cowpea protein isolate	Casein	25	Hypercholesterolemic adults
Hosseinpour‐Niazi, Mirmiran, Hedayati, and Azizi (2015), Iran	Crossover	Both	8	31	58.1	32.9	Legume‐based TLC diet	Legume‐free TLC diet	81	Type 2 diabetes
Lambert et al. (2017), Canada	Parallel	Both	12	53	44	32.9	Yellow pea fiber^m^	Control wafers	15	Overweight and obese adults
Pavanello et al. (2017), Italy	Parallel	Both	12	50	55	26.6	Lupin protein concentrate	Skimmed milk powder	30	Moderately dyslipidemic adults
Kim et al. (2018), South Korea	Parallel	Both	12	18	52.9	23.8	Vigna Nakashima extract	Placebo tablets	3	Type 2 diabetes
Liu et al. (2018), China	Parallel	Both	4	120	57.5	26.6	EABCF vermicelli, instant powder^n^	Low‐GI diet	117.5	Type 2 diabetes
Bartholomae et al. (2019), USA	Parallel	Both	8	37	31.2	23.9	Mung bean protein	Control biscuit	18	Vegetarian adults
Jalal et al. (2020), Saudi Arabia	Parallel	Both	6	60	44.2	26.4	*Phaseolus vulgaris*	None	107	Kidney stone patients
Wang et al. (2020), China	Parallel	Both	5	120	42.6	28.9	*Phaseolus vulgaris* L. capsules	Maltodextrin capsules	2.4	Overweight and obese adults
Ward et al. (2020), Australia	Crossover	Both	8	48	58	29.0	Lupin‐enriched foods	Wheat‐based foods	45	Type 2 diabetes
Doma et al. (2021), Canada (A)	Crossover	Both	4	73	48.1	25.9	Beans^o^	Cooked white rice	190	Elevated LDL cholesterol
Doma et al. (2021), Canada (B)	Crossover	Both	4	73	48.1	25.9	Beans^o^	Cooked white rice	95	Elevated LDL cholesterol
Escobedo et al. (2021), Mexico	Crossover	Both	4	25	26	27.2	Common bean baked snack	None	32	Dyslipidemic adults
Hosseinpour‐Niazi et al. (2022), Iran	Parallel	Both	16	300	55.3	30.7	Legume‐based DASH diet	Standard DASH diet	67	Type 2 diabetes
Rebello et al. (2022), USA	Parallel	Both	8	36	45	34.1	Bean‐based diet	Low‐energy‐dense potato diet	144	Overweight and obese adults
Shaw et al. (2023), Canada (A)	Parallel	Female	8	28	34.6	NR	Pea powder	Maltodextrin powder	130	Female runners
Shaw et al. (2023), Canada (B)	Parallel	Female	8	28	34.6	NR	Pea powder	Maltodextrin powder	120	Female runners
Jäger et al. (2024), USA	Parallel	Both	12	90	47		White kidney bean extract	Placebo	3	Overweight and obese adults

^a^ Before a main meal rich in carbohydrates.

^b^ (230% energy expenditure) (4 days/week servings).

^c^ (Phaseolus vulgaris).

^d^ Replacement of 15%–20% of daily energy intake with the same amount of energy as the control group.

^e^ 5 meals weekly including 750 mL of pulses.

^f^ (60% lupin flour), intervention (4 × 40), control (4 × 32).

^g^ 2 servings of pulses as a substitution and all other breads and cereals were to be wholegrain.

^h^ 2 capsules of dried aqueous extract of *Phaseolus vulgaris* on each meal.

^i^ Bread, biscuits, and pasta with lupin flour (lupin flour was 25%–40% by weight).

^j^ [4 servings/week of non‐soybean legumes (lentils, chickpeas, peas, and beans)].

^k^ 2 servings/day of lentils, chickpeas, beans, and peas.

^l^ 2 capsules of 50 mg of *Phaseolus vulgaris* dry extract 2 times daily.

^m^ Wafers containing 5 g/serving of yellow pea fiber 30 min before 3 large meals.

^n^ EABCF vermicelli with the same calories for one meal and 2 bags of EABCF instant powder before each of the three meals and chew the EABCF hard candies, two at a time, three times a day.

^o^ 1 cup of bean (black, navy, pinto, dark red kidney, and white kidney).

* F, female.

### 2.5 Quality Assessment and Evidence Grading

The methodological quality of the included randomized controlled trials was independently evaluated by two investigators (AN and MST) using the Cochrane Risk of Bias 2 (RoB 2) tool (Page et al. 2021). This approach assesses potential sources of bias across five domains, including the randomization process, deviations from intended interventions, missing outcome data, measurement of the outcome, and selection of the reported results. Each domain was rated as having a low risk of bias, some concerns, or high risk of bias, following the guidelines of the Cochrane Handbook for Systematic Reviews of Interventions. Any discrepancies between reviewers were resolved through discussion or, when necessary, by consultation with a third investigator (RR).

To evaluate the overall certainty of evidence for each primary outcome, the Grading of Recommendations, Assessment, Development, and Evaluation (GRADE) framework was applied (Chandler et al. 2019). Evidence was judged across the domains of risk of bias, inconsistency, indirectness, imprecision, and publication bias and was subsequently classified as high, moderate, low, or very low certainty. The overall strength and confidence in the pooled results were summarized in a Summary of Findings (SoF) table generated using GRADEpro GDT software (McMaster University, ON, Canada).

### 2.6 Statistical Analysis

All statistical analyses were performed using Comprehensive Meta‐Analysis (CMA) software, version 3.0 (Biostat, Englewood, NJ, USA). ffect sizes were expressed as weighted mean differences (WMDs) with corresponding 95% confidence intervals (CIs). A random‐effects model (DerSimonian and Laird method) was used to account for between‐study heterogeneity. Mean differences were calculated as the post‐intervention mean minus the baseline mean for both the intervention and control groups. When change‐from‐baseline standard deviations (SDs) were not available, they were estimated using the formula:

$$SD_{\text{change}}=\sqrt{SD_{\text{baseline}}^{2}+SD_{\text{final}}^{2}-\left(2\times r\times SD_{\text{baseline}}\times SD_{\text{final}}\right)}$$

where r represents the correlation coefficient between baseline and post‐intervention values. In this analysis, a correlation coefficient (R‐value) of 0.8 was considered (Borenstein et al. 2021). For crossover trials, data from paired analyses were extracted whenever available to account for within‐subject correlations. When paired data were not reported, only results from the first intervention period were included in the meta‐analysis to minimize potential carryover effects, following the recommendations of the Cochrane Handbook for Systematic Reviews of Interventions (Chandler et al. 2019).

Between‐study heterogeneity was quantified using Cochran's Q test and the *I* ^2^ statistic, with *I* ^2^ values of 25%, 50%, and 75% indicating low, moderate, and high heterogeneity, respectively. Potential sources of heterogeneity were further explored through subgroup analyses and meta‐regression. Subgroup analyses were conducted intentionally according to age (< 50 vs. ≥ 50 years), BMI (< 28 vs. ≥ 28 kg/m^2^), and intervention duration (< 8 vs. ≥ 8 weeks), based on the median values of the included studies to ensure balanced stratification.

Sensitivity analyses were conducted by sequentially excluding individual studies to assess the stability of pooled results. Publication bias was evaluated visually using funnel plots and statistically using Egger's regression test and Begg's rank correlation test. When publication bias was detected, the trim‐and‐fill method was applied to estimate its potential impact on the summary effect size.

## 3 Results

### 3.1 Selection and Characteristics of Included Studies

**FIGURE 1:**
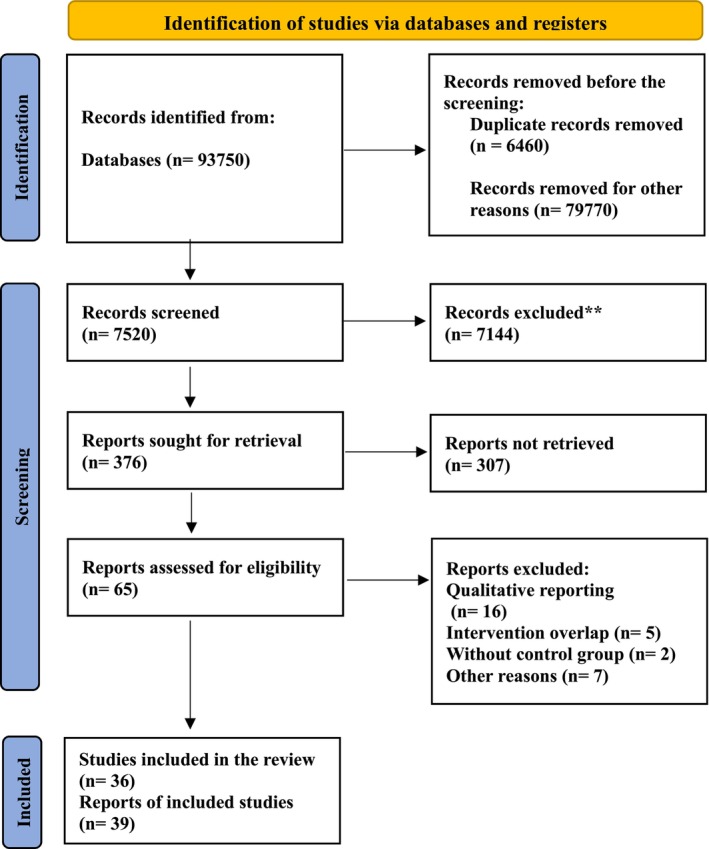
PRISMA 2020 flow diagram for included searches of databases.

In this study, a systematic search was conducted in PubMed (*n* = 15,813), Scopus (*n* = 64,328), and Web of Science (*n* = 13,609). The initial search yielded 93,750 total records. After removing 6460 duplicates and applying our search strategy with keywords, 7520 articles remained for screening. Following title and abstract review and applying inclusion/exclusion criteria, 36 articles were identified as eligible for this meta‐analysis (Celleno et al. 2007; Crujeiras et al. 2007; Winham and Hutchins 2007; Lee et al. 2009; Gravel et al. 2010; Hodgson, Lee, Puddey, Lee, et al. 2010; Venn et al. 2010; Wu et al. 2010; Belski et al. 2011; Hermsdorff et al. 2011; Abeysekara et al. 2012; Jenkins et al. 2012; Turner et al. 2013; Alizadeh et al. 2014; Luzzi et al. 2014; Tonstad et al. 2014; Bähr et al. 2015, 2013; Frota Kde et al. 2015; Hosseinpour‐Niazi, Mirmiran, Fallah‐Ghohroudi, and Azizi 2015; Hosseinpour‐Niazi, Mirmiran, Hedayati, and Azizi 2015; Lambert et al. 2017; Pavanello et al. 2017; Kim et al. 2018; Liu et al. 2018; Bartholomae et al. 2019; Jalal et al. 2020; Wang et al. 2020; Ward et al. 2020; Doma et al. 2021; Escobedo et al. 2021; Hosseinpour‐Niazi et al. 2022; Rebello et al. 2022; Shaw et al. 2023; Jäger et al. 2024; Weiße et al. 2010). The detailed selection process and reasons for exclusion at each stage are presented in the PRISMA 2020 flow diagram (Figure 1).

### 3.2 Study Characteristics and Quality

The included RCTs were conducted across various countries. The study designs consisted primarily of parallel designs, with the following studies employing a crossover design: Winham (Winham and Hutchins 2007), Abeysekara (Abeysekara et al. 2012), Bähr (Bähr et al. 2013), Bähr (Bähr et al. 2015b), Frota Kde (Frota Kde et al. 2015), Hosseinpour‐Niazi (Hosseinpour‐Niazi, Mirmiran, Fallah‐Ghohroudi, and Azizi 2015; Hosseinpour‐Niazi, Mirmiran, Hedayati, and Azizi 2015), Ward (Ward et al. 2020), Doma (Doma et al. 2021), and Escobedo (Escobedo et al. 2021). The dose of non‐soy legume consumption varied across studies, ranging from 0.2 g/day in Luzzi et al. (2014) to 535 mL/day in Gravel et al. (2010). The duration of intervention ranged from 4 weeks in several studies (Turner et al. 2013; Kim et al. 2018; Doma et al. 2021; Escobedo et al. 2021; Bähr et al. 2015) to 72 weeks (Venn et al. 2010).

### 3.3 Qualitative Data Assessment

Table 3 presents the qualitative assessment of the included RCTs. Overall, the methodological quality of the studies was satisfactory. Among the 36 included RCTs, 22 trials were judged to have a low risk of bias, 12 trials had some concerns, and 2 trials were considered at high risk of bias. The most frequent sources of potential bias were related to the randomization process, deviations from intended interventions, and missing outcome data. No major issues were detected in the measurement of outcomes or in selective reporting domains.

**TABLE 3:** Quality assessment of clinical trials (according to Cochrane guideline) investigating the association between non‐soy legumes and body composition.

Study	D1	D2	D3	D4	D5	Overall
Celleno et al. 2007	Some concerns	Low	Some concerns	Some concerns	Low	Some concerns
Crujeiras et al. 2007	Some concerns	High	Some concerns	Some concerns	Low	Some concerns
Winham et al. 2007	Some concerns	High	High	Some concerns	Low	High
Lee et al. 2009	Low	Some concerns	Some concerns	Low	Low	Some concerns
Gravel et al. 2010	Low	Some concerns	Some concerns	Low	Low	Some concerns
Hodgson, Lee, Puddey, Lee, et al. 2010	Low	Some concerns	Low	Some concerns	Low	Some concerns
Venn et al. 2010	Low	Low	Some concerns	Low	Some concerns	Some concerns
Weiße et al. 2010	Low	Low	Low	Low	Low	Low
Wu et al. 2010	Low	Low	Low	Low	Low	Low
Belski et al. 2011	Low	Low	Low	Low	Low	Low
Hermsdorff et al. 2011	Some concerns	Low	Low	Low	Low	Low
Abeysekara et al. 2012a	Low	Low	Low	Low	Low	Low
Jenkins et al. 2012	Low	Some concerns	Low	Low	Low	Low
Bähr et al. 2013	Low	Low	Low	Low	Low	Low
Turner et al. 2013	Low	Low	Low	Low	Low	Low
Alizadeh et al. 2014	Some concerns	Some concerns	Some concerns	Some concerns	Some concerns	Some concerns
Luzzi et al. 2014	High	High	Some concerns	High	Some concerns	High
Tonstad et al. 2014	Low	Low	Low	Some concerns	Low	Low
Bähr et al. 2015	Low	Low	Some concerns	Low	Low	Low
Frota Kde et al. 2015	Low	Low	Low	Low	Low	Low
Hosseinpour‐Niazi, Mirmiran, Fallah‐Ghohroudi, and Azizi 2015	Low	High	Some concerns	Low	Low	Some concerns
Hosseinpour‐Niazi, Mirmiran, Hedayati, and Azizi 2015	Low	High	Low	Low	Low	Some concerns
Lambert et al. 2017	Low	Low	Some concerns	Low	Low	Low
Pavanello et al. 2017	Low	Low	Low	Low	Low	Low
Kim et al. 2018	Low	Low	Some concerns	Low	Low	Low
Liu et al. 2018	Low	Low	Low	Low	Low	Low
Bartholomae et al. 2019	Low	Low	Some concerns	Low	Some concerns	Some concerns
Jalal et al. 2020	Low	Low	Low	Low	Some concerns	Low
Wang et al. 2020	Low	Low	Low	Low	Some concerns	Low
Ward et al. 2020	Low	Low	Low	Low	Low	Low
Doma et al. 2021	Low	Low	Low	Low	Low	Low
Escobedo et al. 2021	Low	High	Low	Low	Low	Some concerns
Hosseinpour‐Niazi et al. 2022	Low	Low	Low	Low	Low	Low
Rebello et al. 2022	Low	Some concerns	Some concerns	Low	Low	Some concerns
Shaw et al. 2023	Low	Low	Low	Low	Low	Low
Jäger et al. 2024	Low	Low	Low	Low	Some concerns	Low

*Note:* D1: Bias arising from the randomization process. D2: Bias due to deviations from intended intervention. D3: Bias due to missing outcome data. D4: Bias in measurement of the outcome. D5: Bias in selection of the reported result.

### 3.4 Effect of Non‐Soy Legume Consumption on Body Weight

**FIGURE 2:**
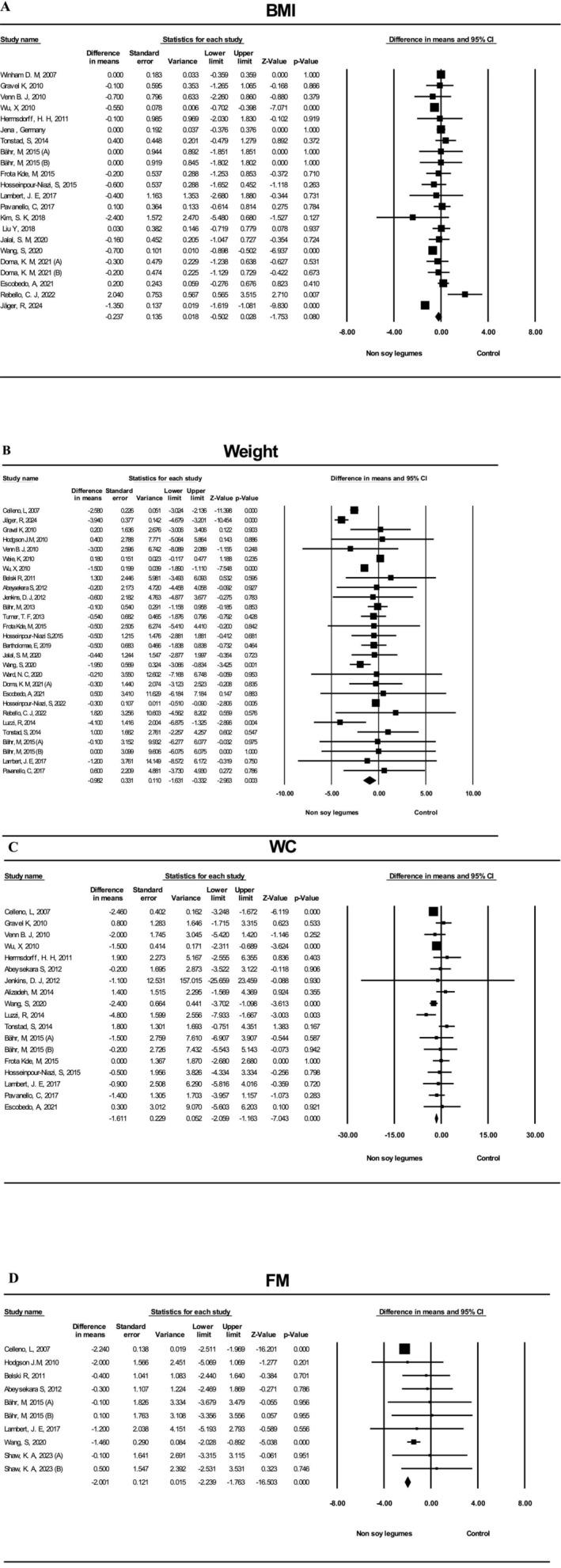
Forest plots of the studied variables. (A) BMI Forest plot. (B) Weight Forest plot. (C) Waist circumference forest plot. (D) Fat mass forest plot.

The meta‐analysis demonstrated a significant reduction in body weight following non‐soy legume consumption compared with control diets (WMD = −0.98 kg; 95% CI: −1.63, −0.33; *p* = 0.003; *I* ^2^ = 87.9%) (Figure 2A).

Subgroup analyses were performed to examine possible sources of heterogeneity (Table 4). In studies with an intervention duration of ≥ 8 weeks, non‐soy legumes significantly reduced body weight (WMD: −0.98 kg; 95% CI: −1.85, −0.10; *p* = 0.02; *I* ^2^ = 85.21%), whereas studies with shorter durations (< 8 weeks) showed a non‐significant change (WMD: −0.87 kg; 95% CI: −2.19, 0.45; *p* = 0.198; *I* ^2^ = 91.66%). Stratification by baseline BMI indicated significant reductions among participants with BMI ≥ 28 kg/m^2^ (WMD: −1.02 kg; 95% CI: −1.92, −0.12; *p* = 0.02; *I* ^2^ = 87.64%), while trials with BMI < 28 kg/m^2^ did not show significant effects (WMD: −0.55 kg; 95% CI: −1.92, 0.81; *p* = 0.42; *I* ^2^ = 90.33%).

**TABLE 4:** The results of the subgroup analysis.

Body weight	> 8 weeks (Group A)	≤ 8 weeks (Group B)	> 28 (Group A)	≤ 28 (Group B)	> 50 (Group A)	≤ 50 (Group B)
No. of comparison	10	18	11	15	16	10
WMD, 95% CI	−0.87 (−2.19, 0.45)	−0.98 (−1.85, −0.10)	−0.55 (−1.92, 0.81)	−1.02 (−1.92, −0.12)	−1.30 (−2.25, −0.35)	−0.29 (−0.49, −0.08)
*p*‐value	0.198	0.02	0.42	0.02	0.007	0.005
*I* ^2^%	91.66	85.21	90.33	87.64	92.01	> 0.001
P. heterogeneity	> 0.001	> 0.001	> 0.001	> 0.001	> 0.001	1.000
P. heterogeneity between group	0.89		0.65		0.12	

BMI	> 8 weeks (Group A)	≤ 8 weeks (Group B)	> 28 (Group A)	≤ 28 (Group B)	> 50 (Group A)	≤ 50 (Group B)
No. of comparison	9	13	12	10	13	7
WMD, 95% CI	−0.21 (−0.57, 0.13)	−0.23 (−0.64, 0.16)	−0.02 (−0.24, 0.18)	−0.36 (−0.74, 0.01)	−0.28 (−0.62, 0.05)	−0.05 (−0.33, 0.21)
*p*‐value	0.22	0.25	0.79	0.06	0.09	0.66
*I* ^2^%	50.48	83.53	> 0.001	84.69	83.41	> 0.001
P. heterogeneity	0.04	> 0.001	0.92	> 0.001	> 0.001	0.72
P. heterogeneity between group	0.94		0.13		0.57	

WC	> 8 weeks (Group A)	≤ 8 weeks (Group B)	> 28 (Group A)	≤ 28 (Group B)	> 50 (Group A)	≤ 50 (Group B)
No. of comparison	7	11	8	9	10	6
WMD, 95% CI	−2.06 (−2.60, −1.43)	−1.13 (−1.77, −0.49)	−1.98 (−2.66, −1.30)	−1.19 (−1.80, −0.59)	−1.81 (−2.30, −1.33)	−0.23 (−1.51, 1.04)
*p*‐value	> 0.001	0.001	> 0.001	> 0.001	> 0.001	0.72
*I* ^2^%	39.14	37.03	> 0.001	51.69	60.77	> 0.001
P. heterogeneity	0.13	0.10	0.49	0.03	0.006	0.91
P. heterogeneity between group	0.39		0.01		0.40	

FM	> 8 weeks (Group A)	≤ 8 weeks (Group B)	> 28 (Group A)	≤ 28 (Group B)	> 45 (Group A)	≤ 45 (Group B)
No. of comparison	4	6	4	4	6	2
WMD, 95% CI	−2.07 (−2.31, −1.83)	−0.48 (−1.58, 0.61)	−2.18 (−2.45, −1.91)	−1.39 (−1.93, −0.86)	−2.04 (−2.28, −1.80)	−0.86 (−2.63, 0.90)
*p*‐value	> 0.001	0.38	> 0.001	> 0.001	> 0.001	0.33
*I* ^2^%	65.19	> 0.001	50.36	> 0.001	61.07	> 0.001
P. heterogeneity	0.03	0.90	0.11	0.77	0.02	0.37
P. heterogeneity between group	0.06		0.37		0.46	

Abbreviations: BMI, body mass index; FM, fat mass; WMD, weight mean difference.

When stratified by age, significant reductions in body weight were observed in both subgroups, with greater effects among participants aged < 50 years (WMD: −1.30 kg; 95% CI: −2.25, −0.35; *p* = 0.007; *I* ^2^ = 92.01%) compared with those aged ≥ 50 years (WMD: −0.29 kg; 95% CI: −0.49, −0.08; *p* = 0.005; *I* ^2^ < 0.001). The marked difference in *I* ^2^ values between the two age groups indicates that age contributed to the observed heterogeneity in the overall analysis.

Meta‐regression analyses (Table S2) showed no significant associations between body weight change and intervention duration (slope = 0.0001, *p* = 0.99), age (slope = 0.05, *p* = 0.21), or BMI (slope = 0.04, *p* = 0.76).

The leave‐one‐out sensitivity analysis indicated that exclusion of any single study did not materially change the pooled effect, confirming the stability of the results. Publication bias assessment using Begg's rank correlation (*p* = 1.00) and Egger's regression test (*p* = 0.53) showed no significant asymmetry (Table S6). The Fail‐Safe N test estimated that 384 additional null studies would be required to nullify the observed effect. The Trim‐and‐Fill method adjusted for potential publication bias by imputing four studies, resulting in an adjusted WMD of −1.08 kg (95% CI: −1.70, −0.45), consistent with the primary analysis.

### 3.5 Effect of Non‐Soy Legume Consumption on BMI

The pooled analysis indicated that non‐soy legume consumption produced a small, non‐significant reduction in BMI compared with control diets (WMD: −0.24 kg/m^2^; 95% CI: −0.50, 0.03; *p* = 0.080; *I* ^2^ = 76.5%) (Figure 2B).

Subgroup analyses were conducted to assess the influence of intervention duration, baseline BMI, and participant age (Table 4). When stratified by duration, the reduction in BMI was not statistically significant for either subgroup (≥ 8 weeks: WMD = −0.23 kg/m^2^; 95% CI: −0.64, 0.16; *p* = 0.25; *I* ^2^ = 83.5%; < 8 weeks: WMD = −0.21 kg/m^2^; 95% CI: −0.57, 0.13; *p* = 0.22; *I* ^2^ = 50.5%). Subgroup analysis by baseline BMI showed a greater, though still non‐significant, reduction among participants with BMI ≥ 28 kg/m^2^ (WMD = −0.36 kg/m^2^; 95% CI: −0.74, 0.01; *p* = 0.06; *I* ^2^ = 84.7%) compared with those with BMI < 28 kg/m^2^ (WMD = −0.02 kg/m^2^; 95% CI: −0.24, 0.18; *p* = 0.79; *I* ^2^ < 0.001). When stratified by age, reductions were more apparent in participants aged < 50 years (WMD = −0.28 kg/m^2^; 95% CI: −0.62, 0.05; *p* = 0.09; *I* ^2^ = 83.4%) than in those aged ≥ 50 years (WMD = −0.05 kg/m^2^; 95% CI: −0.33, 0.21; *p* = 0.66; *I* ^2^ < 0.001). The marked differences in *I* ^2^ values between BMI and age subgroups indicate that both variables contributed to the observed heterogeneity in BMI outcomes.

Meta‐regression analyses (Table S3) revealed no significant associations between BMI change and study duration (slope = −0.008, *p* = 0.54), age (slope = −0.002, *p* = 0.90), or baseline BMI (slope = 0.04, *p* = 0.38).

Sensitivity analysis showed that exclusion of any single study did not meaningfully affect the pooled estimate. Publication bias testing indicated no significant asymmetry, with Begg's rank correlation test (*p* = 0.12) and Egger's regression test (*p* = 0.06) both non‐significant (Table S6). The Fail‐Safe N test estimated that 141 additional null studies would be required to overturn the observed result. After applying the Trim‐and‐Fill method, the adjusted WMD (−0.44 kg/m^2^; 95% CI −0.68, −0.20) remained similar in direction and magnitude to the original estimate.

### 3.6 Effect of Non‐Soy Legume Consumption on WC

The pooled analysis showed that non‐soy legume consumption significantly reduced WC compared with control diets (WMD: −1.61 cm; 95% CI: −2.06, −1.16; *p* < 0.001; *I* ^2^ = 43.1%) (Figure 2C).

Subgroup analyses were performed to assess potential modifiers of this effect (Table 4). When stratified by duration, both shorter (< 8 weeks) and longer (≥ 8 weeks) interventions produced significant reductions in waist circumference (WMD: −2.06 cm; 95% CI: −2.60, −1.43; *p* < 0.001; *I* ^2^ = 39.1%; and WMD: −1.13 cm; 95% CI: −1.77, −0.49; *p* < 0.001; *I* ^2^ = 37.0%, respectively). Analysis by baseline BMI indicated significant reductions in both subgroups, with a larger effect in studies including participants with BMI < 28 kg/m^2^ (WMD: −1.98 cm; 95% CI: −2.66, −1.30; *p* < 0.001; *I* ^2^ < 0.001) compared to those with BMI ≥ 28 kg/m^2^ (WMD: −1.19 cm; 95% CI: −1.80, −0.59; *p* < 0.001; *I* ^2^ = 51.7%). When stratified by age, the reduction in waist circumference was significant among participants aged < 50 years (WMD: −1.81 cm; 95% CI: −2.30, −1.33; *p* < 0.001; *I* ^2^ = 60.8%), while no significant change was observed in participants aged ≥ 50 years (WMD: −0.23 cm; 95% CI: −1.51, 1.04; *p* = 0.72; *I* ^2^ < 0.001). The notable difference in *I* ^2^ values across subgroups suggests that BMI contributed to the observed heterogeneity in waist circumference outcomes.

Meta‐regression analyses (Table S4) confirmed a significant positive association between BMI and changes in waist circumference (slope = 0.30, *p* = 0.0005), whereas duration (slope = 0.001, *p* = 0.95) and age (slope = 0.06, *p* = 0.11) were not significantly related to changes in waist circumference.

Sensitivity analysis showed that removal of individual studies did not substantially alter the overall pooled estimate, indicating stable results. Publication bias was assessed using Begg's and Egger's tests (Table S6). Begg's test showed no significant bias (*p* = 1.00), whereas Egger's regression test indicated mild asymmetry (*p* = 0.04). The Fail‐Safe N test estimated that 50 unpublished studies with null results would be required to eliminate the observed effect. Application of the Trim‐and‐Fill method adjusted for eight potentially missing studies, yielding an adjusted WMD of −2.05 cm (95% CI: −2.47, −1.64), consistent with the main analysis.

The meta‐analysis revealed that non‐soy legume consumption significantly reduced WC (WMD: −1.611 cm, 95% CI: −2.059, −1.163; *p* = 0.001, *I* ^2^ = 43.132), FM (WMD: −2.001, 95% CI: −2.239, −1.783; *p* = 0.001, *I* ^2^ = 49.491), and weight (WMD: −0.982 kg, 95% CI: −1.631, −0.332; *p* = 0.003, *I* ^2^ = 87.890). Reduction in BMI came close to significance, but the change was not statistically significant (WMD: −0.237 kg/m^2^, 95% CI: −0.502, 0.028; *p* = 0.080, *I* ^2^ = 76.482).

### 3.7 Effect of Non‐Soy Legume Consumption on Fat Mass

The pooled analysis showed a significant reduction in fat mass following non‐soy legume consumption compared with control diets (WMD: −2.00 kg; 95% CI: −2.23, −1.76; *p* < 0.001; *I* ^2^ = 65.2%) (Figure 2D).

Subgroup analyses were conducted to investigate potential modifiers of this effect (Table 4). When studies were stratified by duration, a significant reduction in fat mass was observed in trials lasting < 8 weeks (WMD: −2.07 kg; 95% CI: −2.31, −1.83; *p* < 0.001; *I* ^2^ = 65.2%), whereas studies with durations ≥ 8 weeks showed no significant change (WMD: −0.48 kg; 95% CI: −1.58, 0.61; *p* = 0.38; *I* ^2^ < 0.001).

When grouped by baseline BMI, significant reductions were found in both subgroups, with a larger effect in participants with BMI < 28 kg/m^2^ (WMD: −2.18 kg; 95% CI: −2.45, −1.91; *p* < 0.001; *I* ^2^ = 50.4%) compared with those ≥ 28 kg/m^2^ (WMD: −1.39 kg; 95% CI: −1.93, −0.86; *p* < 0.001; *I* ^2^ < 0.001).

Age‐based analysis showed significant reductions among participants < 50 years (WMD: −2.04 kg; 95% CI: −2.28, −1.80; *p* < 0.001; *I* ^2^ = 61.1%), while studies involving participants ≥ 50 years did not demonstrate a significant effect (WMD: −0.86 kg; 95% CI: −2.63, 0.90; *p* = 0.33; *I* ^2^ < 0.001).

The variation in *I* ^2^ across subgroups indicates that duration, age, and BMI contributed to the observed heterogeneity in the pooled analysis.

Meta‐regression analysis (Table S5) supported these findings, revealing significant associations between reductions in fat mass and both age (slope = 0.07, *p* = 0.002) and BMI (slope = 0.26, *p* = 0.003), while duration was not significantly related to effect size (slope = 0.03, *p* = 0.18).

Sensitivity analysis confirmed that omission of individual studies did not meaningfully alter the pooled estimate. Publication bias assessment (Table S6) showed no significant asymmetry by Begg's test (*p* = 0.28), but Egger's regression test indicated potential bias (*p* = 0.003). The Fail‐Safe N test suggested that 134 additional null studies would be required to eliminate the observed significance. After applying the Trim‐and‐Fill method, adjustment for five potentially missing studies yielded a corrected WMD of −2.06 kg (95% CI: −2.30, −1.83 kg), consistent with the original estimate.

### 3.8 GRADE Assessment

The certainty of evidence for the impact of non‐soy legume consumption on body composition outcomes was evaluated using the GRADE approach (Table S7). Overall, the quality of evidence ranged from very low to moderate across the analyzed outcomes. Evidence for reductions in body weight, WC, and fat mass was rated as moderate quality, reflecting consistent findings across randomized controlled trials with no serious risk of bias, but downgraded for inconsistency (*I* ^2^ > 50%) and indirectness due to variations in intervention types and populations. In contrast, evidence for BMI was rated as very low quality, primarily due to serious inconsistency, indirectness, and imprecision resulting from wide confidence intervals and limited study precision. Publication bias was generally not considered a major limitation, although slight asymmetry was noted for WC and fat mass.

## 4 Discussion

In the current meta‐analysis, non‐soy legume consumption significantly reduced body weight, waist circumference (WC), and fat mass (FM), whereas the reduction in BMI was not statistically significant. These findings suggest that non‐soy legumes may improve body composition independent of BMI changes. Subgroup analyses indicated that weight reduction was more evident in interventions lasting longer than 8 weeks and among participants younger than 50 years. In addition, greater reductions in WC and FM were observed in individuals with a baseline BMI < 28, while reductions in BMI among participants with BMI ≥ 28 approached significant but did not reach statistical significance overall.

As mentioned, significant weight reduction was observed in individuals who consumed non‐soy legumes for more than 8 weeks. The observed weight loss of −0.98 kg, while modest, was statistically significant and demonstrates that the intervention can produce measurable changes in body weight. Although larger reductions are typically required for substantial clinical benefits, even modest weight loss can contribute to improved health outcomes over time and may enhance motivation for further lifestyle changes. Thus, this intervention could be a practical and supportive component of a broader strategy for weight management, with the potential for meaningful effects if sustained long‐term. Similarly, J. Venn et al. and Winham et al. reported comparable results in their studies (Venn et al. 2010; Winham et al. 2007). In contrast, the study conducted by Hodgson et al. did not find significant weight reduction, likely due to limitations such as a lack of calorie control, insufficient assessment of physical activity, and the use of variable types of lupins among participants (Hodgson, Lee, Puddey, Sipsas, et al. 2010).

Analysis indicates that the consumption of non‐soy legumes did not significantly decrease BMI. Similarly, a study by J. Venn et al. observed a reduction in BMI; however, this reduction was not statistically significant. Additionally, Hodgson et al. reported a similar insignificant reduction in BMI (Venn et al. 2010; Hodgson, Lee, Puddey, Sipsas, et al. 2010). In contrast, Rebello et al. found a significant reduction in BMI in their study. However, several limitations, such as the absence of a typical Western diet as a control group and notable differences in BMI and fasting insulin levels between the potato and bean groups at baseline, may compromise the validity of their results. Furthermore, the lack of assessment of potential confounding factors, such as physical activity, may also affect their findings (Rebello et al. 2022). Hersmdorf et al. replicated the same significant results based on the same limitations identified in Rebello et al.'s study (Hermsdorff et al. 2011).

Overall, a significant relationship was found between WC and the consumption of non‐soy legumes. After analyzing the subgroups, a significant reduction in WC was observed among individuals who consumed non‐soy legumes for both less than 8 weeks and more than 8 weeks. Furthermore, the reduction in WC was more pronounced in the group that consumed non‐soy legumes for less than 8 weeks. This finding is consistent with several RCTs that also indicate a significant reduction in WC (Gravel et al. 2010; Mollard et al. 2012). In contrast, individuals with a BMI greater than 28 exhibited an insignificant reduction in WC. Additionally, due to limitations such as a small sample size, lack of a control group, and the omission of potential confounding factors, including physical activity and dietary patterns, Karoline de Macêdo Gonçalves Frota et al. reported an insignificant reduction (Hermsdorff et al. 2011).

Analysis revealed a decrease in FM among individuals who consumed non‐soy legumes for a duration less than 8 weeks. Similarly, several RCTs reported similar findings (Hermsdorff et al. 2011). In contrast, Melanie Bähr et al. found no significant reduction in FM, which may be attributed to certain limitations, such as varying adherence to diets and a lack of control over potential confounding factors, including dietary habits (Bähr et al. 2015).

The mechanisms underlying these observed improvements in anthropometric outcomes are not fully elucidated; however, several biological hypotheses may explain these associations. However, several components such as fiber, resistant starch, polyphenols, and L‐Arginine may explain the impact of non‐soy legumes on anthropometric factors. The high fiber and protein content can promote satiety by delaying gastric emptying and increasing fullness, leading to reduced energy intake and subsequent decreases in FM, WC, and weight (Saraf‐Bank and Azadbakht 2015; Tucker 2023). Moreover, the low glycemic index of legumes results in lower postprandial insulin responses compared to high‐glycemic foods. Reduced insulin levels enhance lipid oxidation and decrease fat storage, which may partly account for the reductions in FM and WC (Azadbakht et al. 2011). L‐arginine, a precursor of nitric oxide, may enhance glucose and fatty acid oxidation, thereby promoting fat loss (Azadbakht et al. 2011). Improvements in lipid profiles, including reductions in total and LDL cholesterol levels observed in some trials, may further contribute to metabolic health and facilitate weight regulation (Abeysekara et al. 2012). Polyphenols and other bioactive compounds present in non‐soy legumes may also influence adiposity through antioxidant and anti‐inflammatory pathways (Han et al. 2022). The resistant starch and fiber found in non‐soy legumes can positively influence gut microbiota, which is associated with better energy metabolism and fat oxidation; therefore, weight management, WC reduction, and other beneficial anthropometric outcomes can be achieved. Additionally, non‐soy legumes may influence adiponectin levels, which play a role in regulating glucose levels and fatty acid breakdown. Higher levels of adiponectin are associated with a lower body fat percentage and a reduction in WC (Han et al. 2022; Reyneke et al. 2023). Non‐soy legumes activate peroxisome proliferator‐activated receptors (PPARs), which are involved in lipid metabolism by increasing fatty acid oxidation and reducing fat accumulation (Najjar and Feresin 2019).

All available evidence was gathered on the effects of non‐soy legumes on BMI, weight, WC, and FM. However, several limitations of this meta‐analysis should be acknowledged. First, a relatively small number of randomized controlled trials were available, and substantial heterogeneity was observed in some subgroup analyses, particularly for weight outcomes in interventions lasting less than 8 weeks and for fat mass in longer‐duration interventions. However, heterogeneity was minimal in certain subgroups, such as participants younger than 50 years and waist circumference outcomes in interventions lasting more than 8 weeks, supporting the robustness of these findings. Additionally, considerable variability existed across studies in participant characteristics (e.g., diabetic vs. nondiabetic status, lipid profiles, baseline BMI), study design (parallel versus crossover), sample size, intervention duration, dietary background, and definitions of weight change. The lack of standardization in non‐soy legume type, dose, and dietary context limited comparability and precluded identification of the most effective legume types. Residual confounding related to physical activity, dietary adherence, and other lifestyle factors cannot be excluded. Finally, insufficient data on certain anthropometric outcomes, such as lean mass, prevented further quantitative analyses and highlights the need for additional well‐designed trials.

## 5 Conclusion

Non‐soy legume consumption is associated with favorable changes in several anthropometric indicators, including significant reductions in body weight, waist circumference, and fat mass. These effects were influenced by factors such as intervention duration, participant age, and baseline BMI. However, no significant association was observed between non‐soy legume intake and BMI. Further high‐quality randomized controlled trials are warranted to confirm these findings and to clarify the long‐term effects of non‐soy legumes on overall body composition.

## Author Contributions

**Reza Rahmanian:** writing – original draft, writing – review and editing, visualization, investigation. **Mohsen Shaygantabar:** writing – original draft, writing – review and editing, project administration, supervision, investigation, conceptualization, formal analysis, software, methodology, validation, data curation. **Azita Hekmatdoost:** writing – original draft, writing – review and editing, investigation. **Ali Nikparast:** writing – original draft, quality assessment, writing – review and editing. **Fatemeh Javaheri‐Tafti** and **Zeinab Ghaeminejad:** writing – original draft, screening and investigation. **Andisheh Khoshrang:** screening and data extraction, writing – original draft. **Alireza Hatami:** submission and writing – original draft. **Mohsen Mohammadi‐Sartang:** writing – review and editing and final revision.

## Funding

The authors received no specific funding for this article.

## Ethics Statement

The authors have nothing to report.

## Consent

The authors have nothing to report.

## Conflicts of Interest

The authors declare no conflicts of interest.

## Supporting information


**Appendix S1:** Research strategy.
**Appendix S2:** Meta‐regression for body composition.
**Appendix S3:** Assessment of publication bias in the impact of non‐soy lipid on body composition.
**Appendix S4:** GRADE profile of non‐soy legumes supplementation on body composition.

## Data Availability

The data that supports the findings of this study are available in the Supporting Information of this article.
