# Supplementary material for: Effects of Non‐Soy Legumes on Body Weight and Body Composition: A Systematic Review and Meta‐Analysis of Randomized Controlled Trials
Source: Food Sci Nutr. 2026 Jan 8;14(1):e71365. doi: 10.1002/fsn3.71365 (PMC12783219; doi:10.1002/fsn3.71365)
Supplement: Supplementary file 1 — Appendix S1: Research strategy. Appendix S2: Meta‐regression for body composition. Appendix S3: Assessment of publication bias in the impact of non‐soy lipid on body composition. Appendix S4: GRADE profile of non‐soy legumes supplementation on body composition. [file FSN3-14-e71365-s001.docx]

***Article type:*** systematic review and meta-analysis

**Running title:** Non-soy legumes on body weight and composition: a meta-analysis

**Effects of non-soy legumes on body weight and body composition: a systematic review and meta-analysis of randomized controlled trials**

***Reza Rahmanian^1,2^, MSc****. ORCID ID: 0009-0004-0125-5096*

***Mohsen Shaygantabar^3,4*^, MSc.*** *ORCID ID: 0009-0006-5483-9495*

***Azita Hekmatdoost^5^, MD, PhD, RD.*** *ORCID ID: 0000-0002-1944-0052*

***Ali Nikparast, MSc^3,4^.*** *ORCID ID****:*** *0000-0003-2686-2584*

***Fatemeh Javaheri-Tafti^3,4^, MSc.*** *ORCID ID: 0009-0002-3299-2684*

***Zeinab Ghaeminejad^3,4^, MSc****. ORCID ID: 0009-0002-3299-2684*

***Andisheh Khoshrang^2^, MSc.*** *ORCID ID: 0009-0009-3149-2873*

***Alireza Hatami^6,^****^7^****, MSc.*** *ORCID ID: 0009-0001-8712-0165*

***Mohsen Mohammadi-Sartang^1^, PhD.*** *ORCID ID: 0000-0002-1613-4893*

**^1^** Department of Clinical Nutrition, School of Nutrition and Food Sciences, Shiraz University of Medical Sciences, Shiraz, Iran.

**^2^** Student Research Committee, School of Nutrition and Food Sciences, Shiraz University of Medical Sciences, Shiraz, Iran.

**^3^** Student Research Committee, Department of Clinical Nutrition and Dietetics, Faculty of Nutrition Sciences and Food Technology, National Nutrition and Food Technology Research Institute, Shahid Beheshti University of Medical Sciences, Tehran, Iran.

**^4^** Department of Clinical Nutrition and Dietetics, Faculty of Nutrition Sciences and Food Technology, National Nutrition and Food Technology Research Institute, Shahid Beheshti University of Medical Science, Tehran, Iran.

^5^ Department of Clinical Nutrition and Dietetics, Faculty of Nutrition Sciences and Food Technology, National Nutrition and Food Technology Research Institute, Shahid Beheshti University of Medical Science, Tehran, Iran.

**^6^** Department of Nutrition, Faculty of Medicine, Mashhad University of Medical Sciences, Mashhad, Iran.

**^7^** Student Research Committee, Mashhad University of Medical Sciences, Mashhad, Iran.

**^*^ Corresponding Author**

***Mohsen Shaygantabar***

Department of Clinical Nutrition and Dietetics, Faculty of Nutrition Sciences and Food Technology, National Nutrition and Food Technology Research Institute, Shahid Beheshti University of Medical Science, Tehran, Iran.

Tell: +98-915 801 6058

Fax: +98-915 801 6058

Email: [mohsenshaygan1997@gmail.com](mailto:mohsenshaygan1997@gmail.com)

**Appendix 1. Supplemental Table S1**. Research strategy.

|  | **Search strategy** | **Number** |
| --- | --- | --- |
| **PubMed** | ((Fabaceae[Title/Abstract] OR bean*[Title/Abstract] OR "gram"[Title/Abstract] OR faba[Title/Abstract] OR “pea”[Title/Abstract] OR “peas”[Title/Abstract] OR lentil*[Title/Abstract] OR lupin[Title/Abstract] OR legume*[Title/Abstract] OR chickpea*[Title/Abstract] OR "pulses"[Title/Abstract] OR "non-soy legume"[Title/Abstract] OR "non soy legume"[Title/Abstract]) AND ("randomized clinical trial"[Title/Abstract] OR “RCT”[Title/Abstract] OR "clinical trial"[Title/Abstract] OR random*[Title/Abstract] OR placebo[Title/Abstract] OR intervention[Title/Abstract] OR "double blind"[Title/Abstract] OR "double-blind"[Title/Abstract] OR "cross-over trial"[Title/Abstract] OR “crossover trial"[Title/Abstract]) NOT (soybean*[Title/Abstract] OR soy[Title/Abstract] OR isoflavone*[Title/Abstract])) | 15,813 |
| **Web of Science** | (((TS=(Fabaceae OR bean* OR "gram" OR faba OR “pea” OR “peas” OR lentil* OR lupin OR legume* OR chickpea* OR "pulses" OR "non-soy legume" OR "non soy legume")) AND TS=("randomized clinical trial" OR RCT OR "clinical trial" OR random OR placebo OR "double blind" OR "double-blind" OR "cross-over trial" OR "crossover trial")) NOT TS=(soybean* OR soy OR isoflavone*) | 13,609 |
| **Scopos** | ( TITLE-ABS-KEY ( fabaceae OR bean* OR "gram" OR faba OR "pea" OR "peas" OR lentil* OR lupin OR legume* OR chickpea* OR "pulses" OR "non-soy legume" OR "non soy legume" ) AND TITLE-ABS-KEY ( "randomized clinical trial" OR rct OR "clinical trial" OR random OR placebo OR "double blind" OR "double-blind" OR "cross-over trial" OR "crossover trial" ) AND NOT TITLE-ABS-KEY ( soybean* OR soy OR isoflavone* ) ) | 64,328 |
| **all** |  | 93750 |
| **duplicate** |  | 6460 |
| **Hand screening** |  | 376 |
| **remained for screening title and abstract** |  | 65 |
| **Final for test evaluation** |  | 36 |

**Appendix 2. Supplemental Table S2-5.** Meta-regression for body composition.

**S2. Meta-regression of weight**

| **weight** | **Duration** | **Age** | **BMI** |
| --- | --- | --- | --- |
| **slope** | 0.0001 | 0.05 | 0.04 |
| **CI: 95%lower** | -0.07 | -0.03 | -0.23 |
| **CI:95% upper** | 0.07 | 0.14 | 0.32 |
| **P. value** | 0.99 | 0.21 | 0.76 |

BMI: body mass index, CI: confidence interval

**S3. Meta-regression of BMI**

| **BMI** | **Duration** | **Age** | **BMI** |
| --- | --- | --- | --- |
| **slope** | -0.008 | -0.002 | 0.04 |
| **CI: 95%lower** | -0.03 | -0.03 | -0.05 |
| **CI: 95% upper** | 0.01 | 0.03 | 0.14 |
| **P value** | 0.54 | 0.90 | 0.38 |

BMI: body mass index, CI: confidence interval

| **WC** | **Duration** | **Age** | **BMI** |
| --- | --- | --- | --- |
| **slope** | 0.001 | 0.06 | 0.30 |
| **CI: 95%lower** | -0.05 | -0.01 | 0.13 |
| **CI:95% upper** | 0.06 | 0.15 | 0.47 |
| **P value** | 0.95 | 0.11 | 0.0005 |

**S4. Meta regression of WC**

BMI: body mass index, CI: confidence interval, WC: waist circumference

| **FM** | **Duration** | **Age** | **BMI** |
| --- | --- | --- | --- |
| **slope** | 0.03 | 0.07 | 0.26 |
| **CI: 95%lower** | -0.01 | 0.02 | 0.08 |
| **CI:95% upper** | 0.08 | 0.12 | 0.43 |
| **P value** | 0.18 | 0.002 | 0.003 |

**S5. Meta regression of FM**

BMI: body mass index, CI: confidence interval, FM: fat mass

**Appendix3. Supplemental Table S6.** Assessment of publication bias in the impact of non-soy legumes on body composition.

| **Variable** | **Corrected effect size** | | **Begg's rank correlation test** | | | **Egger's linear regression test** | | | | | **Fail-safe N test** |
| --- | --- | --- | --- | --- | --- | --- | --- | --- | --- | --- | --- |
|  | WMD | 95% CI | Kendall's Tau | z-value | p-value | Intercept | 95% CI | t | df | p-value | N |
| **weight** | -1.08 | -1.70, -0.45 | 0.000 | 0.000 | 1.00 | -0.42 | -1.81,0.96 | 0.62 | 26 | 0.53 | 384 |
| **BMI** | -0.44 | -0.68, -0.20 | -0.23 | 1.52 | 0.12 | 1.17 | -0.07,2.42 | 1.96 | 20 | 0.06 | 141 |
| **WC** | -2.05 | -2.47, -1.64 | 0.000 | 0.000 | 1.00 | 0.98 | 0.34,1.93 | 2.19 | 16 | 0.04 | 50 |
| **FM** | -2.06 | -2.30, -1.83 | -0.26 | 1.07 | 0.28 | 1.38 | 0.59,2.17 | 4.03 | 8 | 0.003 | 134 |

| Quality assessment | | | | | | Summary of findings | | Quality  of evidence |
| --- | --- | --- | --- | --- | --- | --- | --- | --- |
| Outcomes | Risk of bias | Inconsistency | Indirectness | Imprecision | Publication Bias | Number  of intervention/control | WMD (95%CI) |  |
| Body weight | No serious limitations | Serious limitations^a^ | Serious limitations^b^ | No serious limitations | No serious limitations | 1064/1038 | -0.982 (-1.631, -0.332) | ⊕⊕◯◯  Moderate |
| Body mass index | No serious limitations | Serious limitations^a^ | Serious limitations^b^ | Serious limitations^C^ | No serious limitations | 766/804 | 0.237 (-0.502, 0.028) | ⊕◯◯◯  Very low |
| Waist circumference | No serious limitations | No serious limitations | Serious limitations^b^ | No serious limitations | Serious limitations^c^ | 627/625 | -1.611 (-2.059,-1.163) | ⊕⊕◯◯  Moderate |
| Fat mass | No serious limitations | No serious limitations | Serious limitations^b^ | No serious limitations | Serious limitations^c^ | 294/302 | -2.001 (-2.23, -1.763) | ⊕⊕◯◯  Moderate |

**Appendix 4. Supplemental Table S7.** GRADE profile of non-soy legumes supplementation on body composition

^a^ The I2 value was>50% (or Heterogeneity among the studies was high).

^b^ Downgraded for indirectness in the study population.

^c^Publication Bias was detected through Egger and Begg’s test. (p-value<0.05)
